# Supplementary material for: Ten-Year in-Hospital Mortality Trends among Paediatric Injured Patients in Japan: A Nationwide Observational Study
Source: J Clin Med. 2020 Oct 12;9(10):3273. doi: 10.3390/jcm9103273 (PMC7600450; doi:10.3390/jcm9103273)
Supplement: Supplementary file 1 [file jcm-09-03273-s001.pdf]

**Supplementary Materials**

**Table 1.** Missing data by year.

| Variables | 2009 *n* = 917 | 2010 *n* = 1258 | 2011 *n* = 1329 | 2012 *n* = 1637 | 2013 *n* = 1977 | 2014 *n* = 2023 | 2015 *n* = 1977 | 2016 *n* = 1614 | 2017 *n* = 1715 | 2018 *n* = 1621 | *p* value |
|---|---|---|---|---|---|---|---|---|---|---|---|
| Patients with missing data, *n* (%) | 440 (32) | 397 (24) | 558 (30) | 719 (31) | 1312 (40) | 1222 (38) | 1229 (38) | 748 (32) | 700 (30) | 783 (33) | 0.476 |
